# Solid-Phase Microextraction—Gas Chromatography Analytical Strategies for Pesticide Analysis

**DOI:** 10.3390/mps5050082

**Published:** 2022-10-17

**Authors:** Juan Aspromonte, Carlina Lancioni, Giorgia Purcaro

**Affiliations:** 1 Laboratorio de Investigación y Desarrollo de Métodos Analíticos, LIDMA, Facultad de Ciencias Exactas (Universidad Nacional de La Plata, CIC-PBA, CONICET), Calle 47 esq. 115, La Plata 1900, Argentina; 2 Gembloux Agro-Bio Tech, University of Liège, Passage des Déportés 2, 5030 Gembloux, Belgium

**Keywords:** SPME, pesticide, gas chromatography, mass spectrometry, food, environmental

## Abstract

Due to their extensive use and the globalized commerce of agricultural goods, pesticides have become a global concern. Despite the undoubtful advantages of their use in agricultural practices, their misuse is a threat to the environment and human health. Their analysis in environmental samples and in food products continues to gain interest in the analytical chemistry community as they are challenging matrices, and legal concentration limits are particularly low (in the order of ppb). In particular, the use of solid-phase microextraction (SPME) has gained special attention in this field thanks to its potential to minimize the matrix effect, while enriching its concentration, allowing very low limits of detection, and without the need of a large amount of solvents or lengthy procedures. Moreover, its combination with gas chromatography (GC) can be easily automated, making it a very interesting approach for routine analysis. In this review, advances and analytical strategies for the use of SPME coupled with GC are discussed and compared for the analysis of pesticides in food and environmental samples, hopefully encouraging its further development and routine application in this field.

## 1. Introduction

Since its introduction in the early 1990s [1], solid-phase microextraction (SPME) methodologies have been widely explored, proving that it constitutes a non-exhaustive analytical tool which is versatile, appropriate for simple, and effective as sample pretreatment and/or preconcentration step for the analysis of a broad range of analytes in plenty of studies distributed over a wide variety of areas, such as flavors and fragrances, metabolomics, pharmaceutical, and biomedical analysis [2,3,4,5,6]. As an environmentally friendly sampling technology requiring a minimum or zero amount of solvents, it is not surprising that a large amount of papers are devoted to the use of SPME in the analysis of common environmental contaminants, such as polychlorinated biphenyls, polycyclic aromatic hydrocarbons, and pesticides, etc. [7,8,9].

Pesticides are defined as a substance or mixture of substances intended for preventing, destroying, repelling or mitigating any pest; use as plant regulator, defoliant or desiccant or as nitrogen stabilizer [10]. These numerous groups of substances might be categorized according to their origin in natural or synthetic; to their biocide function (i.e., the target pest object) or more frequently, according to their particular moieties in four main types: Organochlorine pesticides (OCPs), organophosphorus pesticides (OPPs), organonitrogen pesticides (ONPs), and pyrethroids [11]. The use of pesticides, mainly in agriculture, is a usual practice that has been spreading worldwide since the mid-20th century to increase crop productivity and preservation, aiming to fulfill the alimentary supply demanded by a continuously growing population [12,13]. Despite the undoubted social and economic advantages of this practice, the vast application of pesticides might produce adverse effects, which can be worsened if good agricultural practices are not satisfied, such as improper handling during the application of these products. On the one hand, a risk of environmental contamination exists. Typically, soil acts as the main pesticide receptor. Indeed, pesticides reach the ground due to a direct soil application or, indirectly, after an application on crops. Once they reach the soil, they will persist and also dissipate toward water bodies and air through wind and rain, leaching, runoff or volatilization. On the other hand, pesticides possess a certain chemical stability and ability to bioaccumulate. Therefore, they represent an important hazard to human health. They may reach the human body by ingesting contaminated drinking water and food (fruit, vegetable, fish, honey, milk, etc.), inhalation of contaminated air or dermal contact with pesticide-contaminated water, air or soil [14].

Considering that this is an issue of global concern, regulatory bodies of multiple jurisdictions have made decisions in this regard, promulgating limit values for pesticides in soil, air, drinking water, and agricultural commodities. The limit values vary in several orders of magnitude depending on the country, pesticide, and matrix. However, they often are as low as parts-per-billon, with some even lower limits [15]. In this sense, the need for accurate, sensitive, and robust analytical methods for pesticide determination at trace levels in a wide diversity of matrices becomes evident. Prior to the instrumental analysis, which is usually a chromatographic method, sample preparation procedures have to be applied to isolate analytes from their matrices, remove interferences, and increase the concentrations to detectable values.

Reported procedures for pesticide analysis usually include the use of liquid-liquid extraction (LLE), solid-phase extraction (SPE), accelerated solvent extraction, and quick, easy, low-cost, effective, rugged, and safe methods (QuEChERS). However, these sample pretreatments are very time-consuming, involve procedures with multiple stages, and present relatively high solvent consumption, which is an attempt against the green chemistry principle of reducing waste. Moreover, these techniques often yield poor analyte enrichment or selectivity, therefore requiring the use of sophisticated analytical instruments to compensate for the lack of selectivity in the sample preparation, such as tandem mass spectrometry (MS/MS), which will require additional maintenance and is only exploited to deal with the matrix.

In this regard, SPME has gained special attention in pesticide determination thanks to its capacity to overcome the abovementioned limitations. Furthermore, compared to liquid chromatography, if SPME is combined with gas chromatography (GC) analysis and thermal desorption as the introduction mode into the instrument, most of the principles of green analytical chemistry are fulfilled [16,17]. Moreover, automatic systems are readily available, increasing their application potential. Therefore, in this review, we focus exclusively on GC. Even though it could be argued that liquid chromatography is the general method of choice for the determination of polar and less volatile compounds, it could be attributed to the compatibility of extracts obtained by traditional preconcentration procedures. At present, with the advances in the development of GC columns and the possibility of tuning analyte chemical properties by derivatization procedures (which can be performed onto the coating), this does not represent an inconvenience if automation and greenness are pondered.

**Figure 1:**
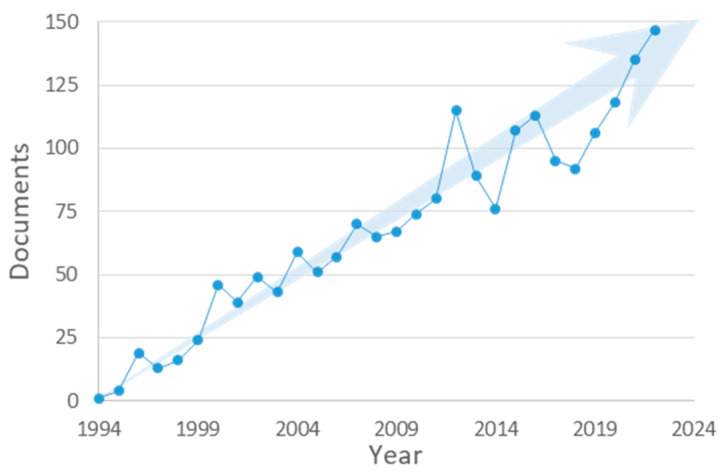
The number of published articles per year from the Scopus bibliometric database [10] using “pesticides”, “gas chromatography”, and “SPME” as descriptors. The number of publications by the end of the current year is extrapolated from the existing data.

When the Scopus bibliometric database [18] is employed to carry out a systematic search within the article title, abstract, and keywords, including the combined descriptors “pesticides”, “gas chromatography”, and “SPME”, the number of published documents by year can be depicted as shown in Figure 1. As can be seen, the interest in using this technique has constantly been growing in this field of application, and it is expected to continue in this direction. A posterior selection of articles to be included in this review was carried out, maintaining those which address the optimization of SPME conditions to some extent.

Despite the numerous reviews on SPME in many fields of applications [19,20], none focus on the advantages of the use of SPME in the cumbersome and highly regulated area of pesticides. In this review, advances in the analysis of pesticides in environmental and food samples using SPME coupled to GC are discussed and compared. Although essential for the betterment of agricultural practices and environmental protection, articles dealing with straightforward applications of the technique without further optimization of the analytical techniques involved, are not considered here. This review is not intended as an exhaustive literature compilation of all the work carried out in this area and characteristic cases were selected to exemplify the different advances in the field. Moreover, the principles of the technique are out of the aim of this review and this information has been largely discussed recently elsewhere [21,22].

## 2. General Considerations

The underlying principle of the SPME methodology is based on the partitioning of analytes between an extractant phase and a sample (liquid or solid). To date, in the fiber approach, the most used geometry which is the extractant phase or sorbent (polymeric liquid, solid sorbent or a combination of both) is deposited on a thin fused silica or metallic rod, which acts as support. Once the fiber is exposed to the gas phase above the sample (HS-SPME) or directly into the sample (DI-SPME), the mass transfer takes place driven by the concentration gradient generated among the two or three phases, respectively. Once the microextraction is performed, desorption is conducted by immersion of the fiber into a compatible solvent or thermally in the injection port of a gas chromatograph.

In SPME method development, it is essential to count with information about the physicochemical properties of both sample matrix and analyte, to set adequate extraction conditions for the attainment of the desired performance of the procedure. Extraction conditions to be established include several variables, such as operation mode, extraction temperature, exposure time, volume phase ratio, pH, ionic strength and pressure, desorption mode, etc. Nevertheless, due to the large number of parameters to be optimized for the SPME and since they could be interdependent, the use of the design of experiments (DoE) should be considered. Combining prior information with simple exploratory designs (Plackett-Burman, Pareto, etc.), the number of parameters that require optimization can be reduced. Then, more complete designs, such as response surfaces, can be used for the remaining ones. However, a key aspect of the overall SPME process relies on sorbent selection. The available marketed sorbents cover a wide range of polarity, volatility, and molecular weight, which are commercialized fibers with single-phase liquid polymeric materials, such as polydimethylsiloxane (PDMS), poly(oxi)ethyleneglycol (PEG), and polyacrylate (PA), as well as mixed-phase materials consisting of solid carboxen (CAR) and/or divinyl benzene (DVB) particles dispersed in PDMS [20,23]. Additionally, research on novel materials development is constantly taking place to improve selectivity and extraction efficiency, reduce costs, increase reusability, enhance porosity and surface area, and provide better chemical, mechanical, and/or thermal stability, etc. Emerging materials employed as SPME coatings include molecularly imprinted polymers (MIPs), metal organic frameworks (MOFs), ionic liquid (IL)-based sorbents, carbon-based materials (graphene, fullerenes, and carbon nanotubes), conducting polymers, monolith-based sorbents and composites [22,23,24]. Concerning pesticide analysis, it can be seen in the following sections that applications involve both commercially available sorbents and novel materials developed for a specific group of pesticides and also to perform multiresidue analysis in different matrices. Details on specific reported works employing novel coatings will be further discussed in following sections.

In addition to the development of new coating materials, there have been advances to increase the sorbent capacity (larger sorbent volumes), while improving the lifespan of the SPME fiber. This has been achieved by changing the geometry of classical fibers into the so-called “Arrow” and HiSorb™ SPME. In the first case, an arrow-shaped metal rod is coated with a sorbent material (similar to those used for classic SPME), obtaining a sorbent volume up to 11.8 µL; this is more than 13 times the volume of a classic 100 µm PDMS fiber (~0.9 µL) [25]. This larger sorbent volume can increase recoveries and improve the extraction capacity of polar compounds, achieving better sensitivity [26]. Moreover, the arrow shape confides a better mechanical stability [27]. In the case of HiSorb™, the idea of increasing sensitivity through a larger sorbent volume has been expanded to obtain 63 µL of sorbent [25]. This came at the cost of considerably enlarging the size of the metal rod, to the point of requiring dedicated modules for desorption, jeopardizing the straightforward compatibility of SPME with GC. Nevertheless, a fully automated platform (Centri) has been developed in 2018 by Markes Int. to overcome this issue [25]. Despite the great potential of these SPME variants, they have not yet been applied to the analysis of pesticides to the best of our knowledge.

Another interesting alternative to increase the sorbent capacity is the use of thin films [28]. These devices are somehow similar to a classic SPME fiber, except that the extraction phase is deposited on a flat substrate, increasing its surface. Its application to the determination of pesticides in water showed promising results for a lab-made device, reaching limits of detection in the range of 20 to 300 ppt when in combination with GC-MS [29]. Nevertheless, the main drawback of these devices is that they are not compatible with GC injector ports, thus they require a compatible thermal desorption system, making their automation less straightforward than the SPME fiber.

Regarding the chromatographic method for pesticide analysis by SPME, there are several reasons to affirm that GC represents a better alternative compared to liquid chromatography. GC possesses better attributes in terms of solvent consumption (greener approach), automation, and cost-efficiency. Limitations in the use of GC for very polar and thermally labile pesticides can be overcome by derivatization procedures and an appropriate column selection [30]. Derivatization can be carried out prior to SPME extraction by adding a derivatizing agent directly into the sample matrix. Then, the derivatized product can be extracted by SPME. Apart from its simplicity, this approach has the advantage of potentially increasing the analyte recovery. For instance, the use of this approach in combination with MS detection allowed limits of detection as low as 2 pg m^−3^ for the determination of pesticides in air [31]. However, possible interferences from the matrix during derivatization should be considered [32]. An alternative to the derivatization prior to SPME, is to use the injection port of the GC to derivatize the analytes after SPME extraction. In this case, a derivatization agent is injected into the GC injection port only prior to the desorption of the SPME fiber. The application of this technique in combination with GC-MS/MS allowed for the attainment of limits of detection in the range of 0.04 to 0.24 ng m^−3^ for the determination of eight pesticides in air. Of note, in these cases, SPME acts as a preconcentration step prior to GC analysis, as the analytes are first collected in air samplers. A third type of derivatization strategy consists of loading a derivatizing agent into the SPME fiber prior to extraction, performing a simultaneous extraction and derivatization on the fiber. This is certainly advantageous in terms of manipulations; however, it may not be simple to find an appropriate derivatizing reagent and the optimization of the process may be cumbersome [33]. Nevertheless, it should be considered that the use of derivatization techniques should be minimized to the greatest possible extent to not hinder the green analytical chemistry potential of SPME [34].

**Figure 2:**
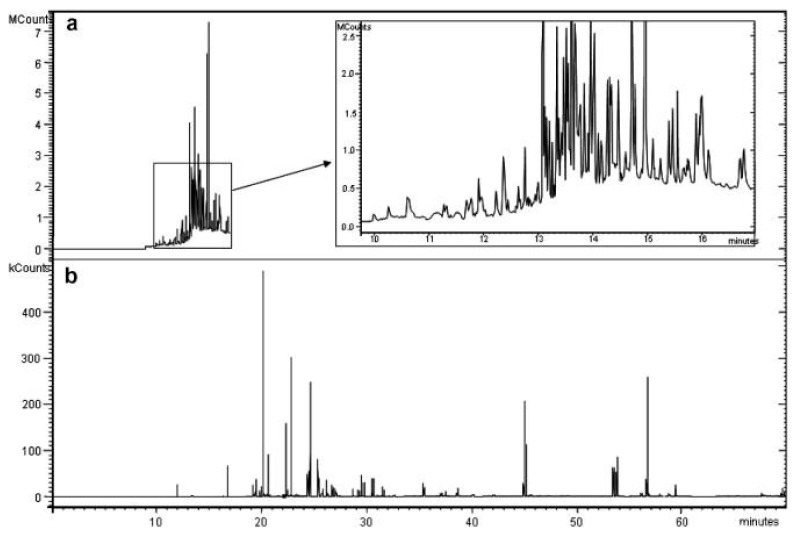
Chromatograms obtained for the analysis by the SPME-GC method of an orange juice spiked with multiple target pesticides at a concentration of 0.05 mg L^−1^ (**a**) obtained with the GC-MS screening full scan method and (**b**) obtained with the confirming/quantifying MS/MS method. Reprinted with permission from Ref. [39]; 2008, Elsevier.

Another important aspect to consider is the detection system to which GC is coupled. Herein, selective detectors exist, which offer good sensitivity and enough specificity toward certain classes of pesticides. For instance, electron capture detection (ECD) is widely used for OCPs, while nitrogen-phosphorus detection (NPD), responsive to compounds containing N and P atoms, is recommended for ONPs and OPPs, respectively. Similarly, the flame photometric detector (FPD), which is sensitive to sulphur- or phosphorous-containing compounds, is also recommended for OPPs analysis. The main drawback of these selective detectors is that their applicability is limited to specific pesticide classes and usually selectivity is not sufficient since there are numerous interfering compounds related to the pesticides of interest, such as transformation products or metabolites. In this sense, MS detection arises as a solution for selectivity and specificity issues. Of note, MS constitutes an extra separation dimension that also allows for the identification of the compounds being separated. However, the use of the full scan mode, rather than selective ion monitoring (SIM) during MS acquisition, guarantee a reliable identification of the analytes, allowing for the performance of untargeted and post-targeted approaches. Nevertheless, the complexity of the samples can hinder this capability and, in particular, can be detrimental for a reliable quantification. For this reason, some authors proposed the use of two stages, first an identification stage using MS in full scan, although the separation of the compounds may not be good for quantification, and then a quantification stage using MS or MS/MS with selected ions for the pesticides that could be identified [35,36,37]. Despite the complexity of the chromatogram obtained for these samples, the use of MS, and, in particular, MS/MS facilitates the quantification of target analytes (predefined or identified during a screening run), enabling multiresidues determinations without a full chromatographic separation (e.g., Figure 2) [35,38,39,40]. When selected ions are used, the identity of the peak can be confirmed by monitoring at least three ions when operating the MS in selective ion monitoring (SIM) or two or three transitions in the case of multiple reaction monitoring (MRM) for MS/MS. For instance, Al-Alam et al. [38] developed a method for the determination of 60 pesticides in honey with limits of detection ranging from 0.12 to 50.42 ppb by means of MS/MS monitoring one precursor ion and one or two product ions for the selected analytes as confirmatory ions.

An interesting alternative to unriddle the complex chromatograms that are obtained for real samples is the use of multidimensional chromatography, which can enable more reliable identification of pesticides through better chromatographic separation. For instance, the use of 2D heart-cut GC allowed for a better resolution of poorly resolved small analyte peaks that overlap with large matrix ones [41,42]. This allowed for identification with a single quadrupole MS of the analytes of interest and it could be used for quantification of the targeted analytes to the sub-ppt levels. Surprisingly, to the best of our knowledge, the combination of the extraction and enrichment capacity of SPME and the use of comprehensive multidimensional gas chromatography (GC×GC) applied to pesticides analysis has been reported only for drinking water [43]. This technique can provide the needed separation power to obtain clearly separated peaks for better identification, facilitating untargeted pesticide screening analysis in challenging samples, such as food and soil, while combining the quantitative enhancement and automation possibilities of SPME.

**Table 1:** Summary of the methods discussed in this review for the two main groups of samples, food and environmental.

Matrix	SPME Coating Tested ^1^	SPME Mode	GC Detector	LOD		Ref.
Food Samples						
Fruits and Vegetables	PA PDMS PDMS/DVB PDMS/CAR DVB/CAR/PDMS	DI	MS (SIM)	1.00–10.00	ppb	[36]
	C-(C3N4@MOF)	DI	MS (SIM)	0.23–7.5	ppb	[44]
	PDMS/DVB	DI	MS (TIC)^2^	0.013–0.110 (for 2D)	ppt	[42]
	PA	DI	MS (identification) FPD (quantification)	0.01–0.14	ppb	[45]
	PDMS (modified)	DI	TOFMS	1–50	ppb	[46]
	COF	DI	ECD	0.04–0.25	ppb	[47]
	PDMS/DVB/PDMS	DI	MS (SIM)	1.0–33.0 only LOQ reported	ppb	[48]
	IL on silica	HS	FID	0.01–0.93	ppb	[49]
	COF	HS	ECD	0.0003–0.0023	ppt	[50]
	PDMS	HS	ECD	0.01–1.0	ppb	[51]
	PDMS PDMS/DVB	HS	MS (TIC) MS (SIM)	0.11–3.48	ppb	[35]
Wine and Juice	PA	DI	MS (SIM) NPD FID	0.01–15 10–6000 200–19000	ppt	[52]
	PA	DI	MS (SIM)	2–90	ppb	[53]
	PDMS PDMS/DVB	DI	MS/MS	0.8–19.6	ppb	[39]
	PDMS/DVB	HS	MS (TIC) ^2^	0.062–33.515 (for 2D)	ppb	[41]
Milk	PDMS/DVB	DI	µECD	0.003–0.56	ppb	[54]
	PDMS PDMS/DVB	DI HS	MS/MS	0.01–0.30	ppb	[40]
	PDMS/DVB	HS	MS (SIM)	2.2–10.9	ppb	[37]
Honey	PA PDMS	DI	MS/MS	0.12–50.42	ppb	[55]
	PDMS PA	DI	AED	0.02–10.0	ppb	[56]
	Electrospun nanostructured PS	HS	MS (SIM)	0.1–2	ppb	[57]
Environmental samples						
Soil and sediment	PDMS	DI	MS (identification) ECD (quantification)	0.6–30	ppb	[58]
	PA	DI	MS (TIC)	0.1–60	ppb	[59]
	PA	DI	MS (SIM) NPD FID	0.01–15 10–6000 200–19000	ppt	[52]
Water (including drinking water)	PDMS/DVB	DI	MS (TIC) ECD NPD	4–32	ppt	[60]
	PA	DI	MS (SIM)	0.05–19	ppb	[61]
	PA	DI	MS (SIM)	3–200	ppt	[62]
	PDMS/DVB	DI	MS (SIM)	0.003–0.322	ppb	[63]
	PDMS/DVB	DI	MS/MS	0.0002–0.04	ppb	[64]
	NU-1000 (MOF)	DI	MS (SIM)	0.011–0.058	ppt	[65]
	DVB/CAR/PDMS	DI	ECD	0.001–0.45	ppt	[66]
	DVB/CAR/PDMS	DI	ECD	0.002–0.070	ppb	[67]
	PDMS/DVB	DI	ECD	2.6–5.7	ppt	[68]
	PDMS	DI	MS ^3^	0.001–0.025	ppb	[43]
	Nafion on SBA-15	HS	MS (TIC)	0.01–0.09	ppb	[69]
	PDMS PDMS/DVB	HS	MS/MS	0.9–26.3	ppt	[70]
	PA	HS	HRMS (magnetic sector)	0.01–350 only LOQ reported	ppt	[71]
	PDMS	HS	ECD	0.034–0.301	ppb	[72]

^1^ PA: Polyacrylate; PDMS: Polydimethylsiloxane; DVB: Divinylbenzene; CAR: Carboxen; MOF: Metal organic framework; COF: Covalent organic framework; IL: Ionic liquid; PS: Polystyrene; SBA-15: Mesoporous silica type SBA-15; ^2^ 2D GC (heart-cut MDGC); ^3^ Comprehensive 2D GC (GCxGC).

## 3. Sample Matrices

Although the determination of pesticides in multiple matrices may be required, the use of SPME as sample pretreatment seems to be focused on environmental and food samples to date. Nevertheless, the determination of pesticides using SPME has also been applied to other matrices, such as human fluids [73,74,75] or agricultural non-food products, such as textiles [76]. Although these applications have shown very promising results, the application of SPME in these areas remains limited when compared to environmental and food applications. The particular interest in these fields is likely due to the regulations imposed by different authorities across the world. In general, pesticides have maximum residue levels allowable in the order of ppb and, even if many of these compounds may be included, no listing can be exhaustive as new variants may be developed and applied despite the local regulations. Therefore, this is a continuously evolving field. SPME has the great advantage of extracting the analytes from the matrix without diluting them, making it possible to reach the needed detection limits, as can be seen in Table 1. Nevertheless, of note, this entails the risk of falsely disregarding some analytes that are not correctly extracted from the matrix by this means. In the following sections, we discuss the applications in food and environmental samples, focusing on the main analytical strategies to attain the required sensitivity.

### 3.1. Food Samples

Agricultural practices had always evolved to cope with the constantly increasing demand for food. The use of pesticides to improve plant growth and pest control have been a crucial development. However, the widespread use of these products entails some potential risks for the environment and health, in long and short terms. Indeed, when pesticides are misused, food products can be a carrier of pesticides into the human body [77]. For this reason, stringent regulations have been in place for some time now [78]. Given that the allowed limits for these contaminants usually are at the ppb level and that food samples are extremely complex matrices, the sample pre-treatment has been a key parameter in the development of new analytical methods in this field. Although classic extraction methods, such as LLE are still in use, they are often combined in multiple steps to enrich the extracts in the analytes of interest effectively. Therefore, techniques that can be very selective in the extraction process have become particularly interesting in this field to reduce matrix interference. This is the case for SPME, in which its potential in this area has been largely explored since its introduction in the early 1990s [79].

In this area, SPME has been implemented in both modes, HS-SPME and DI-SPME. However, the direct immersion mode benefits from higher recoveries, becoming the preferred choice in food matrices. Liquid samples, such as juices [39,53], wines [53] or milk [40,54], can be easily analyzed by DI-SPME. However, they may require some further pre-treatment prior to extraction to minimize matrix effects. For instance, Gonzalez-Rodriguez et al. [40] found poor repeatability and sensitivity when applying DI-SPME to untreated and non-diluted milk samples, which is likely related to the high lipid and protein content of the sample. Fortunately, a simple dilution in water sufficed to overcome the issue. In the case of juices, the reported sampling conditions largely vary between applications. For example, Zambonin et al. [53] reported simple centrifugation and dilution with water prior to DI-SPME extraction, while Cortes-Aguado et al. [39] performed an extraction in ethyl acetate, followed by centrifugation, evaporation to dryness, and dissolution in water:acetone mixture prior to DI-SPME extraction. Therefore, sample pre-treatments and extraction conditions should always be optimized for the intended application.

Other samples, such as vegetables and fruits [36,42,44,45,46,47,48] or honey [38,56] require blending and mixing with diluents to allow for the migration of analytes to the sorbent. Vegetables and fruits are blended and diluted, and in some cases, a centrifugation step is added to further clean-up the matrix. Moreover, in these cases, the extraction conditions vary between applications despite the sample nature. In some cases, the use of small quantities of solvent mixtures are reported [36,42,44,45], while in others, a centrifugation step is included to further clean-up the sample [46,47,48]. Of note, the optimal dilution medium may be different for different analytes even within the same matrix, as reported by Menezes Filho et al. [36]. In the case of honey samples, pre-treatments largely vary without justification, analytes were simply extracted by immersing the SPME fiber into heated buffered aqueous sample solutions of the sample [56] or using a multistep clean-up procedure, including solvent extraction (with small solvent amounts), centrifugation, and concentration by evaporation and redissolution in solvent [38]. Although in the second case 60 analytes were studied (vs. 16 in the other case), the clean-up steps prior to DI-SPME are more cumbersome. Therefore, the pre-treatment should be carefully considered depending on the intended application.

Despite the sample under study, clean-up procedures prior to DI-SPME extraction and extraction conditions require careful consideration. Therefore, parameters, such as pH, temperature, time, and ionic strength are commonly explored during optimization. Of note, the effect of the ionic strength does not always have a significant benefit for the extraction process when working in DI-SPME mode [39,40]. Indeed, the sensitivity may be increased (up to a specific ionic strength) for some analytes by the salting-out effect, while it may be reduced for others. Although samples may require a clean-up step prior to the extraction by DI-SPME, it should be noted that this step remains less labor intensive than conventional techniques, such as LLE. Moreover, even when organic solvents are needed, they are used in very small amounts compared to other techniques, making DI-SPME a greener alternative.

In the case of HS-SPME, the matrix contamination is further minimized and the obtained chromatograms tend to be less complex. Although this mode limits the pesticides that can be analyzed to those that present good affinity toward the gas phase, the sensitivity of these analytes is increased, as observed by Menezes Filho et al. [36]. The same variety of samples as reported for DI-SPME are reported using HS-SPME, namely, fruits and vegetables [35,36,49,50,51], juices [41], honey [57], and milk [37,40,54]. The sample pre-treatment is not significantly different from the ones for DI-SPME. Fruits and vegetables are first homogenized by blending, then centrifugation and filtration may be applied to further clean-up the sample, although this is less needed than for DI-SPME. Thereafter, in all samples, usually, a small amount of solvents, including a brine solution, is added prior to HS-SPME extraction. Of note, this may not always be the best approach, for instance, Rodrigues et al. [37] found that the salting-out effect was detrimental in the recovery of organophosphorus pesticides in milk. 

The HS-SPME extraction is usually carried out by heating the sample to release the analytes from the matrix to the HS. Therefore, the extraction temperature and time have become fundamental parameters in this case, and require careful optimization. It is not surprising to find rising edge situations in response surfaces toward higher temperatures [37]. However, there is always a physical limitation of the maximum temperature a sample may withhold prior to degradation. Although using vacuum as an alternative has been proposed in the area [80], its use is not widespread, and to the best of our knowledge, it has not been reported for pesticide analysis in food matrices.

Due to the highly diverse nature of food products, the extraction procedure optimization must be carried out in a case-by-case approach. Therefore, the comparison of different SPME fiber coatings that are commercially available [35,36,38,40,42,48,54,56] or that are specifically developed for a certain application [44,47,50,57] is often reported. Moreover, the use of experimental designs to obtain optimal conditions has gained popularity, which is likely thanks to the simple to use of software to process the data. Using response surface models after exploring the effects of different extraction parameters, such as temperature, time, pH, ionic strength, etc. has become a common practice [35,37,44,46,48,49,54]. 

Whether it is DI-SPME or HS-SPME, GC is largely chosen as the analytical separation technique. This is mostly thanks to its simplicity for automation when using SPME fibers and the good compatibility with the analytes that are extracted by SPME. Indeed, thermal desorption is largely used to bring the sample into the system thanks to the simplicity and straight compatibility of the SPME fibers with the splitless injector. On the other end of the GC, depending on the target analytes and the application possibilities, different detection techniques are employed. Due to the low levels that need to be detected, ECD [47,50,51,54] and MS [35,36,37,38,39,40,41,42,44,45,46,48,52,53,57] are the most reported detection techniques for this type of samples. Regarding the calibration strategy for quantification, it has to be emphasized that it needs to account for the complex and diverse matrices found in food samples. Standard mixtures for the optimization and validation of the methods, and for quantitation are easily available in set mixtures. These standard mixtures can be used to spike samples of interest or blank samples. In many cases, food samples from organic farming are considered to be free of pesticides and are used as matrix blanks [36,37,38,40,44,45,47,48,50,51]. However, these are not certified materials (at least not for research purposes) and they should be screened to confirm that they are really pesticide free. As aforementioned, the use of multidimensional analysis, such as GC×GC, could be helpful for these untargeted screenings.

### 3.2. Environmental Samples

The rapid increase in the use of pesticides for agricultural practices produced a markedly social concern about the levels of these active ingredients in the environment. As a consequence, the residual levels allowed for these substances are being regulated toward values extremely lower in several matrices, including environmental ones, which include water and soil. Along with these regulations, there is a high demand for analytical methodologies for accurate pesticide determination at trace concentration levels, which overcome the disadvantages of traditional procedures. Namely, long preparation time and large quantities of solvent consumption, as well as laboratory-generated waste. In this regard, SPME has gained special attention and its combination with chromatographic techniques hyphenated to mass spectrometry has been widely used for identification and quantification due to both mentioned pretreatment and separative techniques providing significant improvement in sensitivity and selectivity. Therefore, more extensive research in the area is highly encouraged.

Soil and sediment samples represent challenging matrices since their composition includes a wide variety of minerals, humidity content, and organic matter. The latter acts as a sorbent to which analytes are strongly bonded, hindering the extractive process. As a result, classical method approaches, including Soxhlet extraction, supercritical fluid extraction, accelerated solvent extraction, ultrasonic extraction, and microwave-assisted extraction were selected primarily as pretreatment procedures to desorb analytes from these types of samples. Fortunately, since their introduction, SPME has grown notably and advances related to sorbent development, automation, and SPME-assisted technologies allowed the technique to be considered as an alternative and improved tool for soil and sediment analysis. In these matrices, both DI and HS approaches have been reported for the determination of pesticides and mainly for other environmental contaminants [52], considering two protocols: (i) DI of the fiber in the sample and (ii) preparation of a suspension of the solid matrix by addition of water, brine or a mixture of solvents followed by SPME fiber exposure to the HS or by DI into the liquid phase. On the one hand, the first approach avoids the use of solvent, but prevents the correct diffusion of analytes to the sorbent due to the impossibility of agitating. As a result, microextraction may not be representative of the whole sample. On the other hand, the second strategy provides better extraction efficiencies since the suspension aids in the analyte release from sample pores and allows homogenization by agitation. In this approach, HS is preferred compared to DI since the lifespan of fiber is prolonged.

Even though the application of SPME in soil sampling focused on pesticide determination is scarce, there are some examples in the literature [52,58,59,72,81,82] covering general workflows on which most reported methods are based. For example, Bouaid et al. [58] reported a method based on SPME for the determination of atrazine and four organophosphorus pesticides (parathion-methyl, chlorpyriphos, methidation, and carbophention) in sandy soil samples. Different experimental variables (extraction time, concentration of sodium chloride solution, and desorption time and temperature) were optimized using a central composite design. The final procedure consisted of a previous extraction of the analytes from a weighed amount of soil sample with a small volume of organic solvent (methanol) subjected to ultrasonic agitation. After that extraction, solid particles were separated from the supernatant by centrifugation. A small aliquot of the extract was diluted with deionized water, and this aqueous solution was used for SPME after the addition of NaCl to increase extraction efficiency by the salting out effect. SPME microextraction was performed at ambient temperature by DI of a 100 µm PDMS fiber into the solution under magnetic stirring. Extracted compounds were introduced into a GC by thermal desorption in a splitless operated injector. Analyses were performed in two parallel systems, using an ECD in one case and MS detection in the other. The MS was used in SIM mode, with two fragments for each compound for confirmation. It should be noted that operating the MS in this way solved interferences observed when using the ECD. However, as only two ions were monitored, no confident identification would have been possible [83]. Limits of detection achieved in real soil samples were in the range of 0.6–7 ng g^−1^ except for atrazine and methyl parathion which were 30 and 2 ng g^−1^, respectively.

Similarly, Chang et al. [72] developed a HS-SPME method to determine 10 OCPs in surface estuarine sediments. The optimized procedure consisted of preparing a slurry by placing a weighed amount of sediments previously sieved, deionized water containing a surfactant, and a magnetic bar into a sealed vial. Thereafter, SPME was performed at 70 °C by exposure of 100 µm PDMS fiber to the HS for 60 min while continuously agitating the slurry. Finally, desorption was conducted thermally in a GC injector. Separation was carried out in a gas chromatograph equipped with an ECD. Quantification was performed by analysis of aqueous standards over the range of 0.2–4 ng g^−1^. The developed procedure was compared with Soxhlet extraction using a certified sample, demonstrating good analytical performance and a clear agreement between both methods. 

Regarding water samples, the composition may be as diverse as soil samples, depending on its origin and location. Variable amounts of dissolved organic matter, presence of solid particles, suspended sediments, non-aqueous phase liquids, as well as dissolved gases and inorganic ions may be present. This variability can be attributed to the diversity of water samples which includes groundwater; river, lake, and seawater; influent and effluent wastewater; tap water; ice cores; and snow samples. Even though drinking water may be considered as a food sample, it is reasonable to include it in this section due to matrix similarity. Official methods for isolation and/or preconcentration of target pesticides and other pollutants are based on LLE and SPE [84,85]. In addition to the abovementioned drawbacks related to the multiples stages, time, and solvent consumption, LLE has the disadvantages of being laborious, requiring additional steps for clean-up and concentration by evaporation, and often lead to emulsion occurrence due to the presence of surfactants in the sample. Regarding the SPE approach, negative aspects rely on the need for previous stages of centrifugation or filtration to avoid cartridge clogging. In addition, SPE capacity may not be sufficient, yielding insufficient sensitivity. Therefore, it is not surprising that SPME appears as a powerful and improved technique for sample pretreatment. In this area, a vast number of publications are available [58,59,60,61,62,63,64,65,66,68,69,70,71] dealing with many classes of pesticides in different types of samples. Moreover, non-commercially available fibers with new coating materials were developed for these applications. As presented in Table 1, the results showed good performances with very low LOD in the range of ng L^−1^ or below. Quantification in water analysis by SPME is usually carried out by external calibration prepared with deionized water spiked with pesticides standard solutions and extracting them with the same procedure as the sample. In the majority of the reviewed papers, the determination of pesticides is carried out by GC introducing extracted analytes by thermal desorption, employing mainly MS, ECD, and NPD detection, although other detection systems, such as FID have also been used.

Concerning the SPME mode, HS or DI modes have been reported. For example, Gong et al. [65] have recently reported the fabrication of a novel SPME fiber based on NU-1000 (a zirconium-based metal-organic framework) to be employed for DI extraction of seven OCPs applied to pond and river water. The analytical performance of this fiber coupled with GC-MS (SIM mode) was evaluated under optimal conditions. Briefly, the methodology consisted of filtering water samples and immersing the fiber into a sample aliquot maintained under vibration. DI-SPME optimized conditions were extraction time of 30 min, extraction temperature of 40 °C, desorption time and temperature of 6 min and 260 °C, respectively. Furthermore, the developed fiber was compared with 65 um PDMS/DVB and 85 µm PA fibers commercially available fibers yielding extraction efficiencies that were 3–10 and 2–20 times higher, respectively. 

Domínguez et al. [71] developed a method to simultaneously determine 15 pesticides and other commonly found pollutants in water samples at ultra-trace levels, such as polycyclic aromatic hydrocarbons, polychlorinated biphenyls, and brominated diphenyl ethers. The proposed method based on a combination of HS-SPME and gas chromatography was coupled to high-resolution mass spectrometry in wastewater samples. The authors optimized the extraction procedure and calculated LOQs from matrix-matched calibrations, ranging from 0.01 to 350 ng L^−1^. This approach allowed an increase in the selectivity of the extraction method, largely reducing the potential interferences caused by high molecular mass and other non-volatile interferences present in the sample matrix. At the same time, the lack of direct contact between the fiber and the sample helped in protecting the fiber coating from damage and extended the lifetime of fibers.

Even though HS-SPME is preferable over DI-SPME to prevent fiber damage, it is worth mentioning that an additional step to release analytes from the sample to the HS has to be incorporated prior to uptake by the fiber coating. Consequently, an extra equilibrium is involved in the overall process and extraction efficiencies may be impaired, requiring, therefore, strategies to achieve analyte transport to the HS in operatively reasonable times, especially for analytes with low Henry’s constants. To overcome this limitation, strategies including cold-fiber SPME approach [21,86], vacuum-assisted HS-SPME [86,87], as well as ultrasonic [88], microwave-assisted HS-SPME [89] or multiple-cumulative trapping SPME [90,91] could be applied.

## 4. Concluding Remarks

The analysis of water and food samples to control the presence of pesticides for environmental monitoring or to ensure that products are safe for consumption has become of major interest. Considering the large number of compounds that need to be tested, multiresidue and untargeted pesticides analysis are fundamental to ensure an appropriate determination of all pesticides that can be present. Otherwise, some analytes may be wrongly disregarded, creating a potential environmental and health risk. Given the high complexity and variety of samples, pre-treatment techniques are needed to deal with the complex matrices. SPME has gained the attention of researchers for its simplicity and efficiency in these cases. Indeed, SPME requires only a minimal sample quantity with a simple dilution with small amounts of solvents, often aqueous, to extract multiple analytes of interest in a single-step extraction, making it also a green analytical technique. Moreover, using fibers makes it easy to handle, and the whole process can be automated, contributing to its acceptance for routine applications. Nevertheless, as the sample matrix is almost unique in each case, optimization is required for each sample and different coatings should be tested to find the best solid phase material. On the one hand, this gives rise to new materials that can improve the extraction capacity and be more specifically engineered for these applications. On the other hand, the design of experiments for the optimization of the extraction procedure should be implemented to obtain meaningful and reproducible results. Moreover, different solid phases should be tested, as some analytes may not be adequately extracted due to poor recoveries with certain phases.

As pesticides are a large variety of molecules, multiresidue screening is required, even untargeted analysis should be considered to avoid falsely disregarding some pesticides. Therefore, the use of MS for identification has become unavoidable. Nevertheless, even after SPME extraction, these analyses are targeted and MS is used as an extra separation dimension due to the complexity of the chromatograms obtained and the low detection limits required, losing the full MS identification capacity as it is necessary to rely on confirmation that is derived from the ratio of specific fragments. Therefore, the high separation power of multidimensional chromatography, along with the increased sensitivity obtained thanks to the band compression that occurs in the modulator, could be an interesting approach to obtain better resolved chromatograms that allow for the use of MS in scan mode to identify the different components. Unfortunately, only a very limited number of publications explored this possibility, although its potential has been made evident in other fields.

In conclusion, we hypothesize that the need for pesticides analysis will continue to grow and that SPME and GC will continue to play a central role. New and more efficient materials for SPME may be developed and the use of the design of experiments for optimization of the extraction procedure should be encouraged along with the development of new SPME extraction strategies. Finally, the great potential benefit of multidimensional chromatography, and, in particular, comprehensive techniques, such as GC×GC, should be considered to overcome the challenges posed by the need for a reliable multiresidue screening analysis.

## Data Availability

Not applicable.
